# Developing the Persian Version of Sensory Gating Inventory and Assessing Its Validity and Reliability

**DOI:** 10.32598/BCN.9.10.360

**Published:** 2019-09-01

**Authors:** Mehrnaz Mohebbi, Saeid Mahmoudian, Seyed Abbas Motevalian, Leila Janani, Mohammad Farhadi, Ahmad Daneshi

**Affiliations:** 1. Head & Neck Research Center, Department of Ear, Nose, and Throat Diseases, Iran University of Medical Sciences, Tehran, Iran.; 2. Department of Otorhinolaryngology, Hannover Medical University (MHH), Hannover, Germany.; 3. Department of Epidemiology, School of Public Health, Iran University of Medical Sciences, Tehran, Iran.

**Keywords:** Sensory Gating (SG), Questionnaire, Translation, Perceptual abnormalities

## Abstract

**Introduction:**

Sensory Gating Inventory (SGI) measures behavioral aspects of Sensory Gating (SG), which filters irrelevant sensory inputs into the higher cortex. It modifies sensitivity to sensory stimuli. Abnormal SG leads to overloading of information in the brain and its subsequent dysfunction. Electrophysiological techniques cannot assess the behavioral aspects of SG. We aimed to design the Persian version of SGI with high validity and reliability.

**Methods:**

After a forward and then backward translation of the original SGI, we assessed the content validity and construct validity of the Persian version. A total of 405 participants filled the Persian version of SGI. To assess test-retest reliability, 100 participants filled the inventory again 7–10 days later. The content validity ratio and index, as well as confirmatory factor analysis, were computed, too. Finally, the Cronbach’s alpha, Cohen’s kappa, and intraclass correlation coefficients were calculated.

**Results:**

The content validity ratios of all items of the inventory were more than 60%, which means that they were necessary according to the experts’ opinions. Confirmatory factor analysis confirmed the fitness of the 4-factor structure of the original Inventory. The test-retest reliability using the intraclass correlation coefficient and Cronbach’s alpha coefficients were also high for the four subscales. The Cohen’s kappa coefficients revealed moderate to substantial level of agreement between the first and second scores for all items.

**Conclusion:**

The Persian version of SGI has good and acceptable psychometric properties. It can be used as a valid and reliable tool for studying behavioral aspects of SG in Persian speaking population.

## Highlights

- The SGI is a self-report questionnaire
- The SGI measures behavioral aspects of sensory gating
- The Persian version of SGI was designed and validated
- The Persian version of SGI has acceptable psychometric properties.

## Plain Language Summary

Sensory Gating (SG) is a filtering mechanism of the central nervous system that prevents information processing of unrelated sensory inputs at the higher cortex. SG is necessary for the study of both pathological and normative psychological conditions. It helps the brain to modify its sensitivity to incoming stimuli. SG can be measured behaviorally by Sensory Gating Inventory (SGI). SGI is a self-report questionnaire developed initially by Hetrick et al. (2012). It assesses the neurological aspects of SG in the behaviors of healthy or neurologically/psychologically abnormal people. The SGI has 36 items that address a broad range of perceptual abnormalities related to SG deficit. This study aimed to design the Persian version of SGI (P-SGI) and evaluated its psychometric properties. The content validity ratios of all items of the inventory were more than 60%. The internal consistency and intraclass correlation coefficient for all subscales were high, and kappa values showed moderate agreement. These tests confirmed the reliability of P-SGI.

## 1. Introduction

Sensory Gating (SG) is a filtering mechanism of the central nervous system that prevents information processing of unrelated sensory inputs at the higher cortex (Braff & Geyer, 1990). Normal SG is essential for healthy brain function. SG is necessary for the study of both pathological and normative psychological conditions. It helps the brain to modify its sensitivity to incoming stimuli (Adler et al., 1998). The abnormal function of SG may overload information in the higher cortex and its subsequent dysfunction, which is accompanied by psychiatric symptoms and behavioral disorders (Mcghie & Chapman, 1961).

SG can be measured electrophysiologically by the P50 paradigm (Cromwell et al., 2008) or behaviorally by Sensory Gating Inventory (SGI) (Hetrick et al., 2012). The P50 mid-latency auditory evoked response is the standard electrophysiological index of SG, where a paired-tone paradigm auditory stimulus is used. Also, N100 and P200 have been currently employed with the same paradigm of stimuli to show late phase SG in electrophysiology (Rentzsch et al., 2008) N100 and P200 components in a dual-click procedure. Since P50 sensory gating deficits have been observed in schizophrenic patients and first degree relatives, this parameter was suggested as an intermediate phenotype of the disease. However, most studies only show a low reliability for P50 sensory gating and neither N100 nor P200 sensory gating have been sufficiently tested. METHODS Reliability of P50, N100 and P200 sensory gating was measured in 41 healthy subjects in two sessions, four weeks apart, using intra-class correlation.

Sensory gating was calculated as ratio-gating (second response magnitude/first response magnitude ×100. SGI can represent the clinical features of SG (Micoulaud-Franchi et al., 2015). This questionnaire was developed to objectify the perceptual abnormalities, as well as functional, and psychosocial consequences of SG function. Besides the conventional psychometric and electrophysiological assessments, SGI provides the daily experiences of sensory gating. It also helps to conduct studies on brain-behavior relationships and to assess behavioral aspects of SG - something that P50 cannot. In other words, it clarifies the association between SG underlying mechanisms and the subsequent subjective experiences.

SGI is a self-report questionnaire developed initially by Hetrick et al., (2012) for studying SG in schizophrenic English speakers. With its behavioral questions, it assesses the neurological aspects of SG in the behaviors of healthy or neurologically/psychologically abnormal people. The SGI is composed of 36 items that address a broad range of perceptual abnormalities related to SG deficit. Each item is scored based on a 6-point Likert scale (from 0= never true to 5= always true). The items were grouped based on four influential factors: perceptual modulation, distractibility, over-inclusion, and fatigue-stress modulation. It has strong reliability and validity.

The inventory has already been translated and validated in French (Micoulaud-Franchi et al., 2014) and Japanese (Nobuyoshi et al., 2016). SGI can confirm anomalies of sensory gate and perceptual inundation in schizophrenia (Micoulaud-Franchi and Vion-Dury, 2013; Micoulaud-Franchi et al., 2014; El-Kaim et al., 2015), Attention Deficit Hyperactivity Disorder (ADHD) (Sable et al., 2012; Micoulaud-Franchi et al., 2015, 2016), and Tourette syndrome (Sutherland Owens et al., 2011).

The advantages of the inventory include its low cost, availability, ease of use, the impartiality of scoring/interpretation, and similar questions for all subjects, which facilitate clinical comparison and inference. Thus, it is useful to provide a Persian version of SGI consistent with Iranian culture.

This study was performed based on the International Quality Of Life Assessment (IQOLA) protocol, aiming to create the Persian version of SGI (P-SGI). The inventory should have high validity and reliability to be used in Persian-speaking countries according to their formal language and culture.

## 2. Methods

### 2.1. Study participants

A total of 405 native Persian speakers voluntarily participated in the study. They were 18–59 years old, with no brain injury, current or past substance abuse or dependency, hearing impairment, and neurological diseases. They responded to the final validated P-SGI in Google Forms.

### 2.2. Study procedure

The final validated P-SGI was created in Google Forms, and its URL link e-mailed or sent through social media to the participants. Also, a letter was sent containing the URL link to each participant with the following information: Participating in this study is voluntary; they are free to reveal their names; they will not be compensated for their participation and data, and if they reveal their names, the information will be kept confidential and will not be published in any report. The participants had to register their age, gender, education, and history of any physical and or mental diseases. In case they miss any items of the inventory, the participants had to answer all items of P-SGI.

The subjects rated the items of P-SGI on a 6-point Likert scale (from 0= never true to 5= always true). The algebraic sum of items scores was calculated to obtain the overall score of P-SGI and the scores for each subscale.

### 2.3. Persian translation of SGI

First, the permission of original SGI authors was obtained for translating SGI into Persian. A forward-backward translation was done according to the IQOLA protocol. Two Persian native speakers performed forward translation from English to Persian. They were highly qualified in both English and Persian. The translators also prepared some alternative translations for some words, and finally, they agreed upon Persian version of the inventory. Then, it was checked by a Persian linguist in terms of clarity, quality, conceptual equivalence (similarity of content), and the use of everyday language.

An English native speaker with high proficiency in Persian performed back-translation into English. Then, the differences between the back-translation and the original English version were recognized. We discussed these differences and some words with the authors of the original SGI to select the best alternative words for some items. Table 1 presents the final version of the Persian SGI.

**Table 1.** Frequency and mean scores for each P-SGI item

**No.**	**Items**	**Perceptual Modulation (%)**						**Mean**±**SD**

		**0**	**1**	**2**	**3**	**4**	**5**	
1	Every now and then, colors seem more vivid to me than usual	61.2	24.4	7.9	4.7	1.2	0.5	0.96±0.62
2	Sometimes I find it difficult to focus on one visual sight to the exclusion of others	25.2	29.4	24.4	15.6	4.7	0.7	1.47±1.19
5	At times, I have felt that sounds are flooding me	41.2	30.6	11.9	9.1	5.4	1.7	1.28±1.12
7	Sometimes it seems like someone has turned the volume up — sounds seem really loud	37.3	25.9	18.5	11.4	5.4	1.5	1.29±1.26
8	There are days when indoor lights seem so bright that they bother my eyes	44.4	26.2	12.6	11.9	4.2	0.7	1.24±1.07
10	I hear sounds, but I cannot make sense of them all because it is like trying to do 2 or 3 jobs at once	41.0	23.0	12.8	11.4	9.1	2.7	1.46±1.33
11	For several days at a time, I have such heightened awareness of sights and sounds that I cannot shut them out	53.3	21.5	11.6	7.2	5.7	0.7	1.25±0.93
12	It seems like I hear everything at once	50.6	30.6	8.4	6.4	3.0	1.0	1.11±0.83
14	It seems like I take in too much	27.4	31.1	16.3	14.3	7.4	3.5	1.54±1.39
18	My hearing is so sensitive that ordinary sounds become uncomfortable	57.3	25.7	9.4	4.2	2.5	1.0	1.06±0.72
19	It is not bad when just one person is speaking, but if others join in, then I cannot pick it up at all. I just cannot get into tune with that conversation	45.5	28.4	11.9	4.9	5.9	3.5	1.36±1.08
20	Sometimes I notice background noises more than usual	28.9	35.6	15.1	11.9	6.9	1.7	1.38±1.29
24	I have feelings of being flooded by visual experiences, sights, or colors	64.9	22.2	9.1	3.0	0.5	0.2	0.84±0.53
26	There have been times when it seemed that sounds and sights were coming in too fast	53.6	23.0	12.8	6.7	3.5	0.5	1.14±0.85
27	I cannot focus on one sound or voice and exclude others	26.9	37.8	16.8	10.1	6.4	2.0	1.37±1.26
29	The background noises are just as loud or louder than the main noises	39.0	31.6	15.1	8.4	4.4	1.5	1.22±1.12
**Distractibility**								
3	I find it hard to concentrate on just one thing	24.2	29.6	22.2	14.1	7.7	2.2	1.58±1.31
6	Sometimes I cannot concentrate with even the slightest sounds going on	13.8	31.1	22.2	16.8	12.3	3.7	1.94±1.36
13	I am easily distracted	9.1	30.9	25.2	15.6	14.1	5.2	2.10±1.36
16	It is hard to keep my mind on one thing when there is so much else going on.	8.1	23.2	24.0	19.0	19.8	5.9	2.37±1.39
17	When I am in a group of people, I have trouble listening to one person.	28.6	34.3	17.5	9.6	7.4	2.5	1.40±1.32
22	I find it difficult to shut out background noise, and that makes it difficult for me to concentrate.	17.0	29.1	23.2	13.8	12.6	4.2	1.88±1.40
28	At times, I have trouble focusing because I am easily distracted.	12.6	28.6	24.0	15.8	13.3	5.7	2.06±1.41
31	I have more trouble concentrating than others seem to have.	39.0	24.2	14.6	9.4	8.9	4.0	1.37±1.48
**Over-Inclusion**								
4	The silliest little things that are going on interest me.	9.6	30.1	25.7	16.3	12.1	6.2	2.10±1.37
9	I notice background noises more than other people do.	28.4	30.4	16.5	9.9	10.4	4.4	1.57±1.47
21	Not only the color of things fascinates me, but all sorts of little things, like markings on the surface, attract my attention, too.	15.6	28.6	20.0	18.8	11.4	5.7	1.99±1.43
23	I seem always to notice when automatic appliances turn on and off (like the refrigerator or the heating & cooling system).	12.3	33.6	16.3	15.8	13.8	8.1	2.10±1.50
32	Maybe it’s because I notice so much more about things that I find myself looking at them for a longer time.	31.1	30.6	19.8	7.7	8.9	2.0	1.39±1.33
33	Everything draws my attention even though I am not particularly interested in any of it.	32.1	34.3	13.3	10.4	10.4	8.1	1.33±1.33
34	I seem to hear the smallest details of the sounds.	32.1	31.1	16.8	9.1	8.1	2.7	1.38±1.37
**Fatigue-Stress Vulnerability**								
15	When I am driving at night, I am bothered by the bright lights of oncoming traffic.	9.9	23.5	16.5	19.5	18.0	12.6	2.50±1.56
25	When I am tired, the brightness of lights bothers me.	19.8	24.7	18.0	18.0	10.4	9.1	2.02±1.56
30	I cannot focus on visual images when I am tired or stressed.	10.4	28.9	24.4	18.5	13.3	4.4	2.09±1.34
35	When I am tired, sounds seem amplified.	25.2	29.6	15.3	12.3	12.8	4.7	1.72±1.51
36	It seems that sounds are more intense when I am stressed.	23.5	25.9	18.5	16.3	12.3	3.5	1.79±1.45

### 2.4. Content and construct validity

To assess the content validity of the Persian SGI, ten experts, including neuroscientists, audiologists, and psychologists familiar with the SG concept, rated the necessity of each item through a 3-point descriptive scale. Relatedness, clarity, and simplicity of each item were assessed through a 4-point descriptive scale. Then the Content Validity Ratio (CVR) and Content Validity Index (CVI) of the inventory were computed. To analyze the construct validity, we performed the Confirmatory Factor Analysis (CFA) to test the 4-factor structure of the original SGI. To confirm the fitness of the factor structure, we require a Comparative Fit Index (CFI) greater than 0.9, a Root Mean Square Error of Approximation (RMSEA) less than 0.08, and a Standardized Root Mean Square Residual (SRMR) less than 0.08.

### 2.5. Reliability

The SGI reliability was assessed using internal consistency, interrater, and test-retest reliability. The internal consistency was assessed for each subscale by calculating its Cronbach’s alpha coefficient. Coefficients of more than 0.7 were the cutoff value to confirm the internal consistency of each subscale. For test-retest reliability, 100 participants filled out the inventory again 10 to 15 days later. Then, the test-retest reliability was assessed across the first and second filling of the SGI. The scores of each subscale and the total score were used to calculate the Intraclass Correlation Coefficient (ICC). The interrater reliability was calculated by weighted Cohen’s kappa coefficient to determine the possible agreement between the test-retest scores for each item of Persian SGI. Kappa values <0 indicate no agreement, 0–0.20 slight, 0.21–0.40 fair, 0.41–0.60 moderate, 0.61–0.80 substantial, and 0.81–1 almost perfect agreement (Landis & Koch, 1977). Finally, the effect of independent variables, such as gender, was calculated.

### 2.6. Statistical analyses

Frequencies, means, and standard deviations for SGI total scores, and each subscale were calculated. Also, the construct and content validity was analyzed for the validation process. Test-retest reliability was assessed. The obtained data were analyzed in SPSS V. 21, PASW Statistics and R Statistical software. Stata 11 was used to calculate weighted Kappa’s coefficients.

## 3. Results

### 3.1. Participants’ characteristics

A total of 405 participants, 134 males (33.1%), and 271 females (66.9%), aged 18–58 years (Mean±SD: 27.57±7 y), filled the Persian version of SGI. Considering gender as an independent variable, an independent t-test revealed no significant differences between the two genders regarding the mean score of each subscale and also total scores of P-SGI (P>0.05). The Mean±SD obtained score of P-SGI was 53.93:28.13 (53.25:28.56 in females and 55.14:27.38 in males). Table 2 compares the total score between the two gender groups.

**Table 2.** Independent t-test comparing the Mean±SD of total scores and the score of each subscale for P-SGI between the two genders

**SGI Score**	**Mean**±**SD**		**t**	**P**

	**Female**	**Male**		
Total	53.29±28.49	55.13±27.28	0.62	.53
Perceptual modulation	16.96±12.57	17.77±12.26	0.61	.53
Distractibility	14.43±8.08	15.25±8.37	0.95	.34
Over-inclusion	11.60±6.85	12.36±7.17	1.03	.30
Fatigue-stress vulnerability	10.28±5.74	9.74±4.98	−0.93	.35

### 3.2. Content validity

For checking the content validity, ten experts familiar with the concept of SG rated the necessity of the items by using a 3-point scale (necessary, useful, and not necessary). The minimum reasonable CVR was set as 60% for items. All 36 items showed CVR scores higher than 60%, i.e., the experts confirmed the necessity of all items. CVI of the total score of the questionnaire was 0.93. Besides, the simplicity and fluency, relevance, or specificity and clarity or transparency for each item were measured with a 4-point scale. The results of the CVI scores (>0.8) showed that all phrases in the Persian SGI were simple, fluent, and clearly expressed according to the expert panel.

### 3.3. Construct validity

Confirmatory factor analysis indicated an acceptable fit of the 4-factor structure of the original SGI (RMSEA=0.042, CFI=0.982, SRMR=0.066).

### 3.4. Reliability

**Figure 1.**
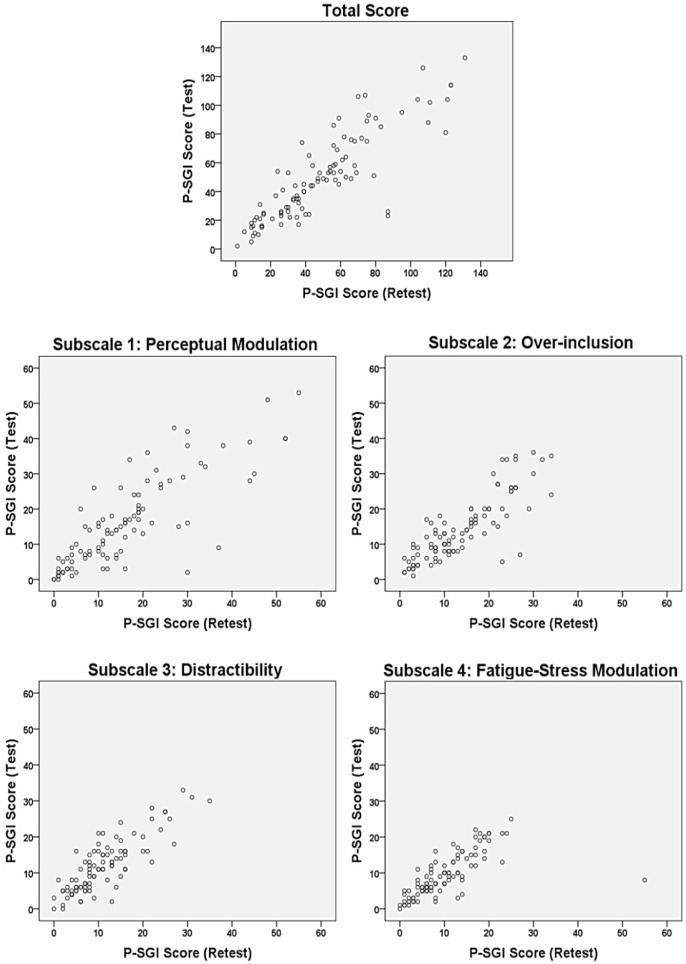
Scatter plots illustrating the relationship between P-SGI scores in test-retest reliability

The internal consistencies of the Persian SGI using the Cronbach’s alpha coefficients were 0.90, 0.88, 0.83, and 0.79, respectively for the four subscales of the inventory. The test-retest reliability was high, showing ICC of 0.91 for all four subscales. The ICC for the total score of P-SGI was 0.93. The scatter plots in Figure 1 show the relationship between P-SGI scores in test-retest reliability. Table 3 presents the values for the ICC and Cronbach’s alpha coefficients. There was moderate to substantial level of agreement between the first and second scores for all items in weighted Cohen’s kappa coefficients (Table 4).

**Table 3.** Values of Cronbach’s alpha coefficient and intraclass correlation coefficient

**SGI Score**	**Mean**±**SD**	**Alpha**	**ICC**	**SEM**
Total	53.87±28.14	-----	0.93	1.39
Perceptual modulation	17.21±12.48	0.90	0.91	0.62
Distractibility	14.69±8.18	0.88	0.91	0.40
Over-inclusion	11.84±6.97	0.83	0.91	0.34
Fatigue-stress vulnerability	10.11±5.50	0.79	0.91	0.27

Alpha: Cronbach’s alpha coefficient; ICC: Intraclass Correlation Coefficient; SEM: Standard Error of Mean

**Table 4.** Values of Cohen’s kappa coefficient for all items of P-SGI

**Cohen’s Kappa Coefficient**	**Items of Persian SGI**
0.4–0.5	2, 6,7, 24, 27, 49
0.5–0.6	1,3,4, 5, 9, 10, 11, 12, 13, 14, 16, 17, 18, 19, 20, 21, 25, 26, 28, 29, 30, 31, 32, 34, 36
0.6–0.7	8, 22, 23, 35
0.7–0.8	15

Values of 0.4–0.6 show moderate agreement; 0.61–0.8 show substantial agreement

## 4. Discussion

The present study provided the Persian version of SGI and assessed its validity and reliability to measure sensory gating in the Iranian population. To the best of our knowledge, this is the first reliability and validation study on P-SGI. The results showed that the items were all necessary, and the CFA analysis confirmed the four subscales of the original SGI (Hetrick et al., 2012) in Persian population. The internal consistency and ICC for all subscales were high, and kappa values showed moderate agreement. These tests confirmed the reliability of SGI.

### 4.1. Gender effect

Comparing the overall and subscales P-SGI scores between the two genders, we found no difference between males and females. Some electrophysiological studies showed less gating for P50 in females compared to males (Hetrick et al., 1996; Patterson et al., 2008) while others found no difference between the two genders in P50, N100, and P200 gating (Lijffijt et al., 2010; White et al., 2005; Freedman et al., 1987; Waldo et al., 1987). Hetrick et al., (1996) suggested that differences in auditory gating between males and females were not because of biological differences in the generators of P50 and N100, instead due to the distinct impact of inhibitory mechanisms affecting the generator of these evoked potentials.

The results of the present study agree with the findings of those electrophysiological studies, which found no difference between the genders. Similar to previous studies, the overall score of P-SGI indicated no effects on gender (Hetrick et al., 2012; Micoulaud-Franchi et al., 2014). In contrast to our findings, Hetrick et al., (2012) found significantly higher scores on “distractibility”, “fatigue”, and “stress vulnerability”, subscales in females than males. They concluded these higher scores are due to greater “distractibility” and “mind wandering” (Giambra, 1980) and more susceptibility to effects of stress and fatigue in women than men. White et al., (2005) demonstrated significantly impaired P50 suppression during the stressor condition in females than males but no significant differences between the genders for N100 suppression under the stressor condition.

Since the participants’ level of stress was not controlled or checked before filling the inventory, we hypothesize that women in the study of Hetrick et al., (2012) may be under more stress during the study, but the female participants in our study were under less stress. Also, the intelligibility of items may affect their scoring. Items such as “distractibility”, “fatigue”, and “stresses” are more susceptible to be affected by stress, tiredness, and concentration. So selecting some types of sentences and their intelligibility can affect their scoring. Persian sentences may be more explicit.

### 4.2. Content validity

The CVI of P-SGI was found to be acceptable. However, the CVR was a bit lower in items 24, 35, and 36 compared to the others. These differences are probably due to the type of selected words and the opinion of experts. Unlike our study, content validity has not been assessed in previous studies (Micoulaud-Franchi et al., 2014; Nobuyoshi et al., 2016). In an investigation of content validity, Polit & Beck (2006) confirmed that the CVI is a simple validity method. It shows the amount to which a sample of items establishes an adequate operational definition of a concept (Polit & Beck (2006).

### 4.3. Construct validity

The results of CFA were compatible with the original scale (Hetrick et al., 2012) and the French SGI (Micoulaud-Franchi et al., 2014). These results show that the 4-factor structures presented by Hetrick et al., (2012) are also appropriate to investigate sensory gating in the Persian population. CFA indicators for P-SGI showed better results compared to French SGI; however, both confirm the factor structures of the original SGI.

### 4.4. Test-retest reliability

Our results suggest that the test-retest reliability of the PSGI using ICC is relatively high. Congruent with our study, Hetrick et al., (2012) indicated good test-retest reliability for SGI using ICC. They assessed the test-retest reliability of SGI at one of three inter-trial intervals across the retest intervals of 4.5, 6.5, and 9 weeks after the first session, but we performed it only once and 10–15 days after the first session. They suggested that the temporal stability of the SGI and its factors is significant within 4–9 weeks in healthy subjects. However, Micoulaud-Franchi et al., (2014) did not conduct-test-retest reliability analyses for French SGI. Of the essential characteristics of a valuable tool are repeatability and consistency of obtained scores (Polit and Beck, 2006). In other words, if someone repeats the same test several times, he or she should get the same results.

### 4.5. Internal consistency reliability

The internal consistency of the P-SGI by Cronbach’s alpha value was very high (0.93), and for all subscales ranged between 0.79 and 0.90. Hetrick et al., (2012) reported moderate-to-large internal consistency reliability of SGI for each of the four subscales ranging from 0.75 (“fatigue” and “stress vulnerability”) to 0.92 (Perceptual modulation). Micoulaud-Franchi et al., (2014) indicated satisfactory internal consistency for all subscales of French SGI, ranging from 0.79 to 0.92. Lower internal consistency on the sub-scale of “fatigue” and “stress vulnerability” was reported in all studies compared with other subscales.

It may be due to the different fatigue and stress conditions of the participants in different sessions of completing the inventory. Fatigue and stress conditions of individuals can affect selecting the scales for each item. So, it may be necessary to revise the items such that the stress has less effect on individuals. However, P-SGI had the highest internal consistency score among SGI of other languages. Our results of internal consistency agree with the previous studies. This result indicates that the response to every item matches the response of the total items in the P-SGI. These findings suggest that P-SGI is a reliable tool for assessing SG.

### 4.6. Interrater reliability

Weighted Cohen’s kappa coefficient showed a moderate to substantial level of agreement between the first and second P-SGI scores for all items. Since all items of P-SGI had moderate to substantially weighted kappa coefficient, changing the content of items was not necessary. Previous studies did not assess the reliability of SGI by kappa coefficient; therefore, we did not have any similar study to compare our results with them.

SG problems may disturb attention and perception (Mcghie & Chapman, 1961). Shortages in SG have been reported in psychological disorders such as schizophrenia (Patterson et al., 2008), bipolar disorder (Sánchez-Morla et al., 2008), ADHD (Holstein et al., 2013), Alzheimer disease (Jessen et al., 2001), obsessive-compulsive disorder (Ahmari et al., 2012) and tinnitus (Rauschecker et al., 2010). SGI has been used in studying some of these diseases and can detect the SG dysfunction in schizophrenia (Micoulaud-Franchi et al., 2014), Tourette syndrome (Sutherland Owens et al., 2011), and ADHD (Sable et al., 2012); thus it is a valuable tool in detecting abnormalities in sensory input perception in psychiatric disorders.

Micoulaud-Franchi et al., (2017) proposed a short version of the SGI. Although the SGI-36 presents high acceptability, it may be too lengthy for patients with inattention symptoms. It is usually recommended that questionnaires for clinical populations should be as brief as possible because they have difficulties in perception and concentration (Ware, 2008). We suggest working on designing short Persian SGI, which seems to be more useful in clinical practices.

## 5. Conclusion

In conclusion, this study demonstrated that P-SGI has good and acceptable psychometric properties. It can be used as a validated and reliable tool for studying sensory gating in Persian speakers.

Regarding the limitations of the present study, we should address the sample size, which was relatively small, and consisted of participants mostly with higher education from the urban area. The validation and reliability process of P-SGI should be performed in less-educated and non-educated individuals and participants from rural areas, too. The gender ratio was in favor of women. We suggest that future studies be conducted on larger sample sizes and participants with low educational levels living in rural areas. We also recommend that other validity methods, such as external validity and criterion-related validation, be performed. Convergent and discriminant validity can provide valuable information about the external validity of the SGI. However, this study provided preliminary information for further research in validity and reliability of P-SGI and future studies on SG in Persian population. Although Google Forms had a question about the history of any physical or mental diseases, some participants might not reveal all or part of their illnesses. Thus, we cannot be sure about the healthy condition of all study participants.

## Ethical Considerations

### Compliance with ethical guidelines

The study procedure was in accordance with the Declaration of Helsinki. The Ethical Review Board of Iran University of Medical Sciences (IUMS) approved the study procedure (Code: IR.IUMS.REC 1396.29494).
